# Anabolic Steroid-Induced Cholestatic Jaundice in a Young Adult: An Emerging Clinical Challenge Underscoring the Importance of a Thorough Drug History

**DOI:** 10.7759/cureus.108860

**Published:** 2026-05-14

**Authors:** Min Kyaw Han, Winluck Shayo, Zaw Thant Lwin, Moe Thazin Aung, Cynthia Cheng

**Affiliations:** 1 Acute Medicine, University Hospitals of Derby and Burton NHS Foundation Trust, Derby, GBR; 2 Neurology, University Hospitals of Derby and Burton NHS Foundation Trust, Derby, GBR

**Keywords:** anabolic-androgenic steroids, bodybuilding supplements, cholestasis, drug-induced liver injury, methandrostenolone, performance-enhancing drugs

## Abstract

Anabolic-androgenic steroids (AASs) are synthetic derivatives of testosterone with both androgenic and anabolic properties, and their non-medical use has been increasingly reported, particularly among young adults. Although licensed for conditions such as hypogonadism and cachexia, AASs are frequently misused for rapid muscle growth and performance enhancement. Such unregulated use carries a significant risk of drug-induced liver injury, most commonly presenting with a cholestatic pattern. Here, we describe a case of a previously healthy young bodybuilder who developed severe cholestatic jaundice after seven weeks of unsupervised anabolic steroid use. This case highlights the importance of thorough drug history taking, including over-the-counter supplements and performance-enhancing agents, in any young adult presenting with unexplained jaundice. Early recognition and prompt withdrawal of the offending agent resulted in marked clinical improvement and steady normalisation of liver function tests.

## Introduction

Anabolic-androgenic steroids (AASs) are synthetic compounds that resemble the male hormone testosterone and exert both anabolic and androgenic effects [1]. While they are medically indicated for conditions such as endocrine disorders (e.g., primary and secondary hypogonadism), haematological conditions (e.g., aplastic anaemia), and catabolic states, including HIV-related muscle wasting, chronic kidney disease, and certain malignancies [2], their non-medical use has increased substantially since their development in the 1930s. This rise is particularly evident among young males seeking rapid improvements in physical appearance and strength, representing a growing public health concern [3].

The non-medical use of AASs has been widely reported across Europe, Australasia, and North America. The Crime Survey for England and Wales reported that approximately 0.2% of adults aged 16-59 (around 59,000 individuals) had used anabolic steroids in 2013 [4]. Many AASs are purchased online or obtained over the counter, often concealed within unregulated dietary supplements marketed for performance enhancement, which pose risks of contamination, mislabelling and undisclosed pharmacologically active substances [5].

AAS-associated hepatotoxicity has been well-documented since the 1950s, with manifestations including cholestasis, hepatocellular injury, hepatic neoplasms, and peliosis hepatis [6]. Given that the liver is the primary clearance site for AASs, hepatotoxicity remains a key adverse effect, with clinical manifestations ranging from reversible dysfunction to severe liver injury, depending on the type of AAS, as well as the dose and duration of exposure [7]. This case report highlights a characteristic cholestatic pattern of drug-induced liver injury associated with 17α-alkylated anabolic steroids, particularly methandrostenolone (Dianabol).

This article was previously presented as a poster at the Royal College of Physicians and Surgeons, Glasgow - Medicine24 Conference on October 23-24, 2025.

## Case presentation

A 30-year-old male bodybuilder with no significant past medical or family history presented to the Medical Same Day Emergency Care (SDEC) unit with jaundice, dark urine, and generalised pruritus. He denied alcohol consumption but reported taking over-the-counter anabolic steroids (Dianabol/methandrostenolone) at a dose of 10 milligrams every 6 hours for 7 weeks (total cumulative dose approximately 1,960 milligrams) for muscle building.

He smoked 15-20 hand-rolled cigarettes daily. Although his maternal grandmother died of liver cancer at age 42, this was considered unlikely to contribute to his acute cholestatic presentation.

On initial assessment, the patient was clinically stable with no signs of acute liver failure. Laboratory investigations showed a cholestatic pattern of liver injury: bilirubin 76 µmol/L (normal <21), alanine aminotransferase (ALT) 238 U/L (normal <40), alkaline phosphatase (ALP) 214 U/L (normal <129), and gamma-glutamyl transferase (GGT) 49 U/L (normal <50). A non-invasive liver screen, including viral and autoimmune serology, was negative, and the abdominal ultrasound was also unremarkable, helping to exclude alternative causes of jaundice. Furthermore, assessment using the Roussel Uclaf Causality Assessment Method (RUCAM) yielded a score of 9, indicating a highly probable drug-induced liver injury [8].

On review 10 days later, he reported worsening pruritus and jaundice. Repeat liver function tests showed further deterioration with bilirubin 138 µmol/L, ALT 129 U/L, ALP 315 U/L, and GGT 29 U/L. Compared with prior results, there was a rise in bilirubin, a reduction in ALT, and a further increase in ALP, consistent with a predominantly cholestatic pattern of liver injury. Given the concern for possible acute liver failure, he was admitted to the Medical Assessment Unit for close monitoring and review by the gastroenterology team. He was diagnosed with drug-induced cholestatic jaundice secondary to anabolic steroid use.

Management included discontinuation of anabolic steroids, cholestyramine powder 4 grams daily, and antihistamines for symptomatic relief. He was discharged after clinical stabilisation, with strong advice to abstain from further anabolic steroid use. He was also counselled regarding warning signs of worsening liver dysfunction, including increasing jaundice, abdominal pain, confusion, bleeding tendency, and worsening pruritus, and was advised to seek urgent medical attention should these occur.

At two weeks post-discharge, his symptoms persisted, and liver biochemistry remained deranged: bilirubin 60 µmol/L, ALT 734 U/L, and ALP 313 U/L. Despite the marked rise in ALT, his GGT remained within the normal range, consistent with anabolic steroid-induced liver injury.

At four weeks of follow-up (six weeks after cessation of anabolic steroids), there was marked clinical and biochemical improvement. His pruritus had completely resolved, bilirubin had normalised to 18 µmol/L, and ALT and ALP had improved significantly to 348 U/L and 233 U/L, respectively, as summarised in Table 1. Continued gradual normalisation of liver enzymes was anticipated with ongoing abstinence from anabolic steroids. As liver function was steadily improving and there were no features suggestive of hepatic decompensation or other red-flag features, the gastroenterology team opted for a conservative watch-and-wait approach. In view of the improving biochemical trend and stable clinical condition, invasive procedures, such as liver biopsy, were not deemed necessary at that stage.

**Table 1:** Liver biochemical trends over time (gradual improvement of liver enzymes with ongoing abstinence from anabolic steroids) µmol/L, micromoles per litre; U/L, units per litre

Time point	Bilirubin (µmol/L)	ALT (U/L)	ALP (U/L)	GGT (U/L)
Presentation	76	238	214	49
Admission (10 days later)	138	129	315	29
2 weeks post-discharge	60	734	313	45
4 weeks post-discharge	18	348	233	44
Normal range	<21	<40	<129	<50

## Discussion

Non-medical use of AASs has extended well beyond competitive athletes and is now common among recreational gym users, typically young males aged 18-35 years [3,4,9]. Their use is largely driven by appearance- and performance-enhancing goals and facilitated by online markets offering easy access to unregulated products. Although the cardiovascular and endocrine complications of AAS misuse are well-known [2,5,6,9], liver injury, particularly the cholestatic pattern associated with 17α-alkylated steroids, such as methandrostenolone (Dianabol), remains under-recognised [7,10-12]. 

Our patient’s symptoms, including progressive jaundice, troublesome pruritus, and dark urine, closely matched the classic presentations described in similar cases [1,7,11-13]. His biochemical profile of marked hyperbilirubinemia, elevated ALP, relatively modest transaminase levels, and normal or near-normal GGT was characteristic of AAS-induced bland cholestasis [1,7,11,13]. Several reports also describe normal imaging findings and preserved GGT levels despite markedly elevated bilirubin concentrations, helping differentiate this pattern from obstructive or inflammatory causes [1,7,11-13]. In addition, the clinical course in our case--delayed rise in bilirubin after stopping anabolic steroids, followed by slow improvement--also mirrors what has been reported in the literature, where recovery may take several weeks to months [1,7,12,13]. This delayed bilirubin peak also highlights the importance of continued monitoring during the recovery phase.

The mechanism of injury is thought to relate to 17α-alkylation that allows oral absorption while increasing hepatotoxic potential. Experimental studies show that AASs can impair bile formation, disrupt the hepatocellular cytoskeleton, and interfere with bile transporter function [10-12]. Furthermore, activation of androgen receptors by AASs not only mediates intrahepatic microfilament damage but also suppresses expression of genes (ATP8B1, ABCB11) involved in bile acid transport and secretion, resulting in bile acid accumulation, cholestasis, and cholestatic jaundice [7]. These mechanisms help explain why patients can develop profound cholestasis with no structural abnormalities on imaging, as seen in our case. Imaging in reported cases, including abdominal ultrasound or CT, is frequently unremarkable, and extensive viral, autoimmune, and metabolic workup often yields no alternative explanation [1,7,11,13].

Management focuses on immediate cessation of the anabolic steroid and symptomatic treatment of pruritus, commonly with cholestyramine. While agents such as ursodeoxycholic acid have been used in prolonged cases, evidence supporting their benefit remains limited, and systemic corticosteroids are not routinely recommended [1,7,12,13]. Regular clinic follow-up and serial monitoring of liver function tests are important to assess recovery and detect potential progression. In our case, the patient responded well to conservative therapy alone, and the predictable improvement in liver biochemistry supported a watch-and-wait approach without the need for biopsy [7,12,13].

This case highlights two practical messages. First, clinicians must ask directly about AAS or supplement use, as many individuals do not disclose it unprompted, and products may not be accurately labelled [3-5]. Second, recognising the distinct cholestatic pattern associated with 17α-alkylated steroids can help avoid unnecessary tests and reassure patients about the expected trajectory of recovery [1,7,12,13]. Given the continued prevalence of AAS misuse [3,4,9], early identification, patient education, and greater public awareness remain key to preventing this fully avoidable cause of jaundice.

## Conclusions

AAS-induced liver injury is an emerging clinical challenge, especially among young adults aged 18-35 years presenting with jaundice or unexplained liver function abnormalities. Diagnosis may be delayed, as patients do not always initially disclose the use of anabolic steroids or performance-enhancing supplements. Early recognition and discontinuation of the offending agent remain the cornerstone of management.

Although most patients improve gradually over weeks to months with supportive therapy alone, ongoing monitoring of liver function during recovery remains important. Given the rising misuse of anabolic steroids driven by gym culture, social media influence, and unregulated online sales, clinicians should routinely enquire about AAS and supplement use when evaluating unexplained cholestasis. Education, open discussion, and a multidisciplinary approach involving primary care, hepatology, and sports medicine are essential to reduce preventable liver injury in this growing population.
